# Enhanced Permeability of Etoposide across Everted Sacs of Rat Small Intestine by Vitamin E-TPGS 

**Published:** 2013

**Authors:** Abdolhamid Parsa, Roonak Saadati, Zahra Abbasian, Saeed Azad Aramaki, Simin Dadashzadeh

**Affiliations:** a *Department of Pharmaceutics, School of Pharmacy, Shahid Beheshti University of Medical Sciences, Tehran, Iran.*; b *Clinical laboratory section, Khatamolanbia hospital, Tehran, Iran.*; c *Pharmaceutical Sciences Research Center, Shahid Beheshti University of Medical Sciences, Tehran, Iran.*; d *Student Research Committee, Shahid Beheshti University of Medical Sciences, Tehran, Iran. *

**Keywords:** Etoposide, Vitamin E-TPGS, Everted gut sac, Permeability, P-glycoprotein

## Abstract

Etoposide, a widely used anticancer drug, exhibits low and variable oral bioavailability mainly because of being substrate for the efflux transporter, P-glycoprotein (P-gp). Therefore, the present study was aimed to investigate the effect of D-*α*-tocopherol polyethylene glycol 1000 succinate (TPGS) and PEG 400 as P-gp inhibitors on the intestinal absorption of etoposide. Everted sacs of rat small intestine were incubated in Krebs buffer solution which contained etoposide in the absence or presence of various concentrations of TPGS or PEG 400. The effect of verapamil as a known P-gp inhibitor on the absorption of drug was also studied.

The absorptive transport of etoposide was significantly enhanced (p < 0.001) in the presence of verapamil (100 μg/mL) and TPGS (over the concentration range of 0.002- 0.1 mg/mL), suggesting that the inhibition of P-gp located in the intestine may be involved in the enhancement of etoposide absorption. However, the addition of PEG 400 at various concentrations (0.05, 0.1 and 0.5% w/v) had no effect on the etoposide transport. No significant difference was found between the permeability values in the absence and presence of the maximum concentration of TPGS for two transport markers, lucifer yellow and imipramine, indicating that the enhancement in etoposide permeability in the presence of TPGS was not due to the compromise in tight junctions or membrane integrity of epithelial cells.

The results of the study suggest that the use of TPGS as a safe excipient in etoposide formulations may enhance the oral bioavailability of etoposide and result in a predictable oral absorption.

## Introduction

Etoposide, a semi-synthetic derivative of the bioactive lignan podophylotoxins widely used, alone or in combination with other chemotherapeutics, to treat a variety of cancers, both of solid tumors and hematological malignancies (1). This anticancer drug exhibits low and erratic oral bioavailabilities (25 ± 75%) with considerable intra- and interpatient variation. Therefore, achieving optimum clinical benefit remains a major concern.

Etoposide has been reported to be a P-glycoprotein (P-gp) substrate (2-4). P-gp, the gene product of MDR1 and a 170 KDa plasma protein, functions as an important membrane transporter and energy-dependent drug efflux pump to decrease drugs and xenobiotics accumulation in a variety of systems (5). Under normal physiological conditions, P-gp is expressed in a wide range of tissues, such as the lung, kidney, liver, adrenal tissues, pancreas, and colon as well as in the brush border membrane of the small intestine (5-8).On the intestinal level, it is located in the apical membrane of the epithelial cells and transports drugs back into the gut lumen. Studies in animals and human have indicated that P-gp plays a major role in limiting drug absorption and consequently oral bioavailability (9, 10). These effects have restricted the clinical use of drugs which are substrates of P-gp. Thus, there is considerable interest in trying to enhance their absorption and oral bioavailability by inhibiting the P-gp-mediated drug efflux.

A P-gp inhibitor agent can overcome the barrier and increase drug absorption. Several chemicals such as verapamil, cyclosporine A and PSC 833 have been proved to be potent P-gp inhibitors and they can improve the bioavailability of a number of valuable drugs (11, 12). But their toxicities due to the pharmacological effects have hindered their use in clinical application (13).

Recently, it has been reported that some excipients, which are largely used as inert vehicles in drug formulations, could inhibit the function of P-gp in the intestine. These excipients (or additives) offer advantages of being safe, not being absorbed from the gut, pharmaceutically acceptable and have a history of being incorporated in many parenteral and enteral formulations as solubilising or stabilizing agents (14). Several studies have demonstrated that some of them may disrupt the function of intestinal P-gp and thus enhance the intestinal absorption of the drugs which are substrates of P-gp. Therefore, they could offer new opportunities to improve the oral bioavailability of clinically useful drugs that are P-gp substrates. Based on these advantages and compared to other P-gp inhibitors, excipients seem to be a better choice. Lo demonstrated that Tween 20, Tween 80, Myrj 52 and Brij 30 increased the epirubicin transport and reduced efflux in diffusion chambers with excised rat intestinal mucosa (15). In other studies pluronic P85 was found to increase the permeability of a broad spectrum of drugs in Caco-2 cell monolayers (16), and it also enhanced drug absorption in the Ussing chamber (17).Furthermore it has been shown that some excipients such as PEG-400, Tween-80, Pluronic F-68 and Cremophor EL-35 could increase the transport amount of ganciclovir in the everted gut sac model (18).

Vitamin E-TPGS (d-α-tocopherol polyethylene glycol 1000 succinate) is a water-soluble derivative of natural source vitamin E and was reported to be non-toxic even at a dose of 1.0 g/Kg/day (19). This compound has been used as a solubilising agent and emulsifier and it has been characterized as an inhibitor of P-gp-mediated drug transport in the human intestinal Caco-2 cell monolayers and other cell lines (20, 21). It has been shown to enhance the oral bioavailability of colchicine in rats (22). Also TPGS increased the *in-vitro *permeability of paclitaxel (23) and celiprolol (24) in Ussing chamber and everted gut sac model, respectively.

In this study, we evaluated the effect of vitamin E-TPGS and PEG-400 on the transport of etoposide in rat small intestine. Therefore we performed the permeability investigation by using the *in-vitro *everted sac model. Effect of two above mentioned excipients on etoposide permeability was evaluated compared to verapamil, as a representative P-gp inhibitor. In addition the effect of TPGS on paracelluar and transcellular rout of absorption was examined.

## Experimental

*Materials*

Etoposide was kindly provided by Cipla (Mumbai, India). Verapamil hydrochloride and imipramine were supplied from Roozdarou and Pars Darou, respectively (Tehran, Iran). HPLC grade acetonitrile and methanol were obtained from Caledon (Georgetown, Canada). Purified water was prepared using a Millipore Direct-QTM (Millipore Corporation, Bedford, MA, USA). Lucifer yellow, D-α-tocopherol polyethylene glycol 1000 succinate (TPGS) and PEG-400 were provided by Sigma-Aldrich (Steinheim, Germany). All other chemical reagents used were of pharmaceutical grade.

*Animals*

Male Wistar rats (240-270 g), obtained from the Pasteur Institute (Tehran, Iran) were maintained in a controlled environment of 25°C with a 12-12 h light/dark cycle. The rats were fasted overnight before experimentation and had access to water ad libitum. All protocols and procedures were approved by the local ethics committee for animal experiments of Shahid Beheshti University of Medical Sciences (Tehran, Iran).

*Preparation of gut sacs*

The everted sac method was used as previously described (25). Everted intestinal sacs were prepared by quickly removing the small intestine from starved rats killed under CO_2_-anesthesia. The jejunum was excised, flushed through several times with saline solution at room temperature and placed immediately into oxygenated buffer solution at 37°C. Then the intestine was gently everted over a steel rod, filled with fresh oxygenated buffer solution and divided into sacs approximately 4.5 cm in length with silk suture. Sacs were preincubated in oxygenated buffer solution at 37°C for 5 min and then placed in 25 mL oxygenated buffer solutions at 37°C containing 25 μg/mL etoposide with or without the P-gp inhibitors in different concentrations. At the defined time points sacs were removed and blotted dry. The sacs were cut open and the serosal fluid was drained into small Eppendorf vial to determine the drug concentration. The area of each sac was calculated closely. Each sac was weighed before and after fluid collection to calculate accurately the volume inside the sac.

*LDH release of the everted gut sac*

The viability and any possible damage of the gut were evaluated by measuring the release of the cytosolic enzyme lactic dehydrogenase (LDH) as an indicator of cell damage (18). The LDH activity was determined in the incubation media in the absence or presence of the excipients using LDH-P kit from Kimia pajouhan (Tehran, Iran). The results were calculated as U/L/cm^2^ of sac area (1 unit reduces 10^-6^ mol pyruvate per min at pH of 7.5). The influence of excipients (using the maximum concentrations) on the viability of gut sac was measured at 30, 60 and 90 min and compared to the control group (without adding any excipient).

*Glucose transport across the everted gut sac*

The viability and the integrity of the gut sacs were further demonstrated by analyzing the glucose concentrations both in the mucosal and serosal sides. Viable enterocytes transport glucose against a concentration gradient, so in a non-leaking metabolically active membrane it should be possible to measure a glucose gradient between the external medium and the serosal fluid (26). Samples of incubation medium and content of the sacs were collected at predetermined times. Glucose concentrations were measured by a kit from Pars Azmoon (Tehran, Iran) and an automated biochemistry analyzer Hitachi 902 (Roche Diagnostics, mannheim, Germany).

*Effects of excipients on drug transport across gut sac*

Sacs were incubated at 37°C, in 25 mL of oxygenated Krebs buffer containing etoposide (25 μg/mL) in absence (control group) or presence of verapamil (100 μg/mL)**, **TPGS (0.002, 0.02 and 0.1 mg/mL) or PEG-400 (0.05, 0.1 and 0.5% w/v). The transport of etoposide from mucosa to serosa was measured by analyzing the serosal medium at 10, 20, 35, 45, 60, 70 and 90 min. The same procedure was performed for imipramine (5 μg/mL) in presence of the highest concentration of TPGS (0.1 mg/mL) as a transcellular marker.

*Analysis of etoposide and imipramine*

Concentrations of etoposide and imipramine were analyzed by HPLC. The HPLC system consisted of a Wellchrom K-1001 HPLC pump, a Wellchrom online degasser, a Rheodyne auto injector equipped with a 100 μl loop, Wellchrom K-2701 UV/VIS lamp, and K-2700 diode array UV/VIS spectrophotometric detector (all from Knauer, Germany). The chromatographic data was acquired by Chromgate 3.1 software from Knauer. Analyses were carried out at ambient temperature on a Chromolith Performance RP-18e (100 mm 4.6 mm *i.d.*, Merck) coupled with a Chromolith RP-18e guard cartridge (5.0 mm 4.6 mm *i.d.*, Merck). The optimal mobile phase for etoposide was a mixture of acetonitrile and purified water (35:65 v/v) and for imipramine was acetonitrile and 25 mM phosphate Buffer (40:60 v/v) .The mobile phase was filtered using a Millipore filter (0.45 mM),degassed by vacuum stirring for 2 min and delivered at a flow rate of 1 mL/min. The detection wavelength was set at 240 nm and 252 nm for etoposide and imipramine respectively, and the injection volume was 100 μL. The calibration curves were linear over the concentration range of 0.25-5 and 0.25-7 μg/mL for imipramine and etoposide, respectively (correlation coefficient of R^2^ > 0.998) and the lower limit of quantification was 250 ng/mL for both drugs). The intra-day and inter-day coefficients of variation (CV) were all less than 6%.

*Analysis of lucifer yellow*

Lucifer yellow was chosen as a fluorescent hydrophilic paracelluar marker to evaluate epithelial cell tightness in this experiment (27). Lucifer yellow was measured by a direct spectrofluorimetric method with excitation and emission wavelengths of 418 and 512 nm, and quantified with a calibration curve prepared in buffer solution. Mucosal (1 mL) and serosal (0.5 mL) samples were centrifuged for 10 min at 4000×g to precipitate mucus and other solid matter and were then diluted 10 and 2 times, respectively.

*Statistical analysis*

The results are expressed as the mean ± standard deviation and were analyzed using one-way ANOVA with post-hoc (Tukey›s test) and considered statistically significant when p < 0.05.

## Results and Discussion

*Determination of etoposide and imipramine*

**Figure 1:**
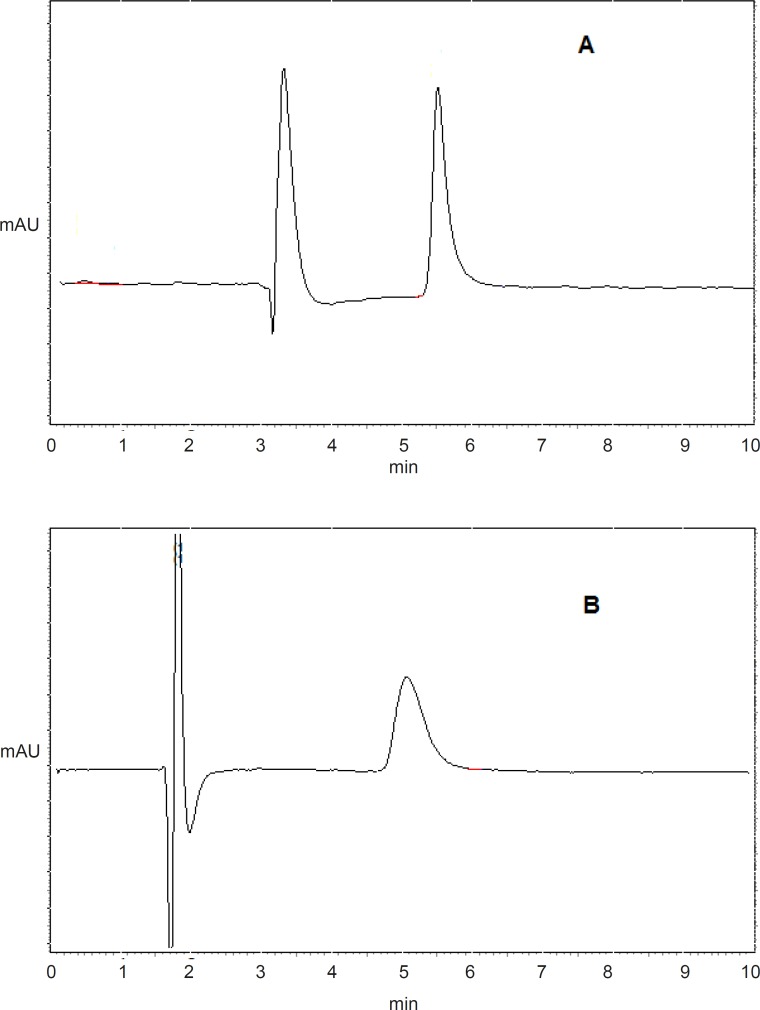
Chromatograms of etoposide (A) and imipramine (B) from serosal medium in everted gut sac model.

Figure 1 shows the typical chromatograms of etoposide (A) and imipramine (B) from serosal medium in everted gut sac model. Retention times were 5.4 min for etoposide and 5 min for imipramine.

*Glucose transport across the everted gut sac model*

**Figure 2:**
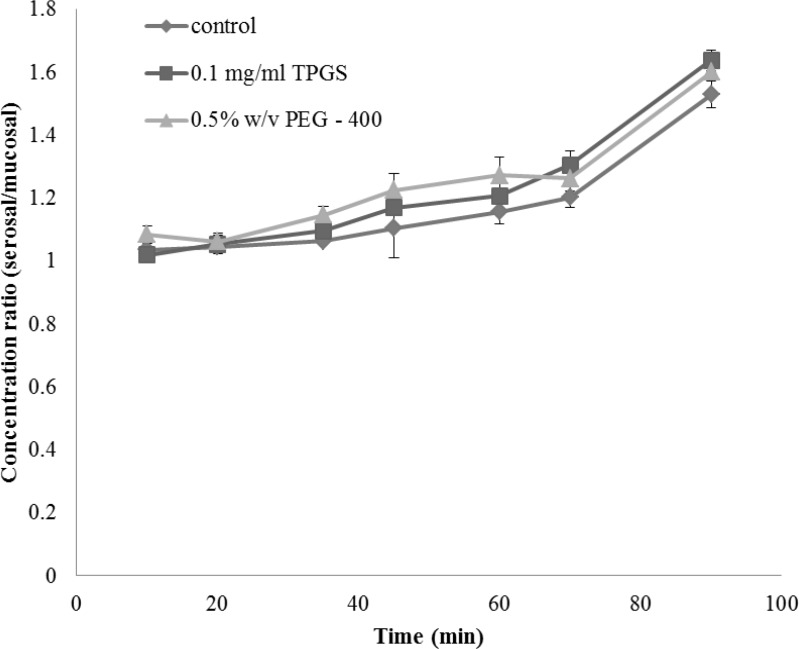
Glucose transport in everted gut sac model. Data are shown as mean ± SE (n = 4).

The integrity of the sacs was confirmed by the active transport of glucose across the membrane from mucosal to serosal side. The concentration ratios of glucose between serosal side and mucosal side in the presence and absence of TPGS are shown in Figure 2. It can be seen that the ratios were gradually increased and reached a factor of about 1.5 at 90 min and addition of TPGS had no obvious effect on the glucose gradient.

As glucose is actively transported in the small intestine, intact and metabolically active sacs will maintain a glucose gradient between the external medium and serosal fluid. As shown in Figure 2 in both control and TPGS treated groups glucose concentration inside the sacs (serosal side) was approximately 1.5 times higher than the outside (mucosal) concentration, indicating that the tissue of gut sac was viable and well functioning.

The active transport of glucose requires metabolic energy and so clearly if the sacs were not biochemically active, or if they were not physically intact, such a concentration gradient would not be maintained (28).

*LDH release in everted gut sac model*

**Figure 3:**
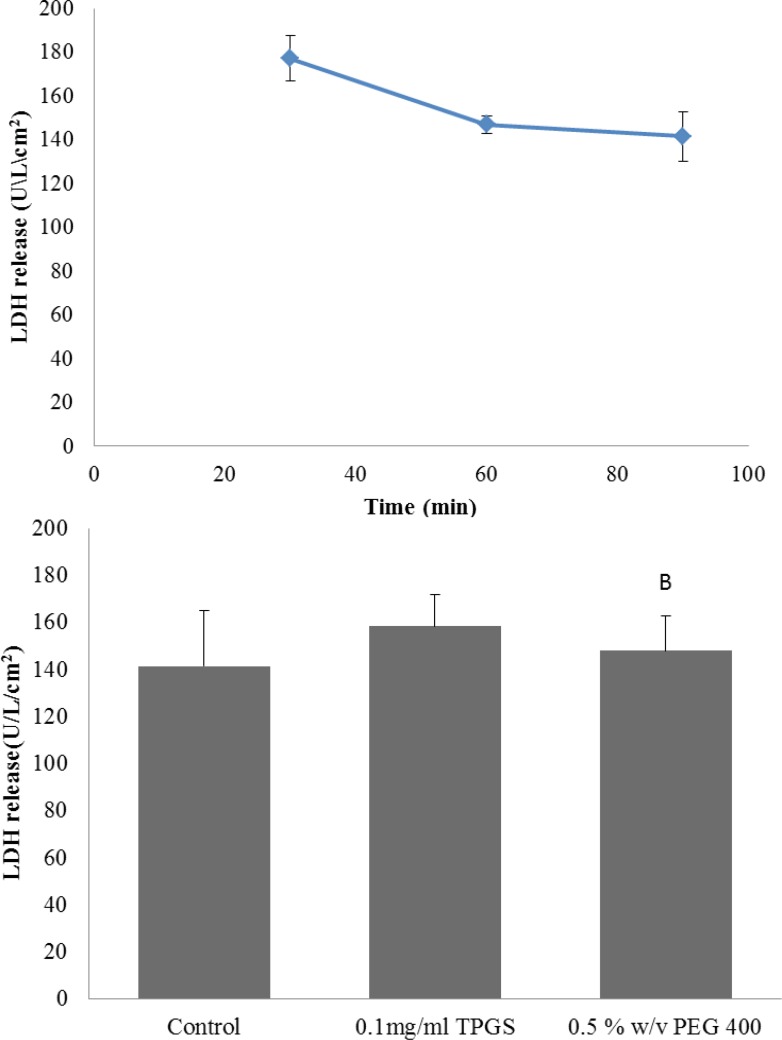
LDH release in everted gut sac model. (A) The time course of LDH release in the control group; (B) LDH release in absence or presence of excipients at 90 min. Data are shown as mean ± SE (n = 3-5).

In order to further evaluate the viability of gut sac and any possible damage due to the sac preparation the release of the cytosolic enzyme LDH was examined. The LDH result for control group is shown in Figure 3 A. At 30 min LDH activity in the incubation media was 177 U/L/cm^2^ and it was not significantly different from the LDH of 60 and 90 min (p > 0.05), suggesting the viability of the gut sacs during the experiments. Also LDH level could be a good index to evaluate the intestinal membrane toxicity, since many toxic substances could stimulate the release of this enzyme (29).Therefore, enzyme level was measured in presence of TPGS 0.1 mg/mL and PEG 0.5% w/v (as the highest concentration of two excipients) as well. It can be seen from Figure 3 B that LDH release were not notably changed by TPGS and PEG-400. Hence, the everted gut sac model was suitable for studying the effect of these excipients on intestinal transport of drugs and the results obtained from an incubation period of 90 min can be regarded as reliable. 

*Effects of excipients on etoposide transport across gut sac*

**Figure 4:**
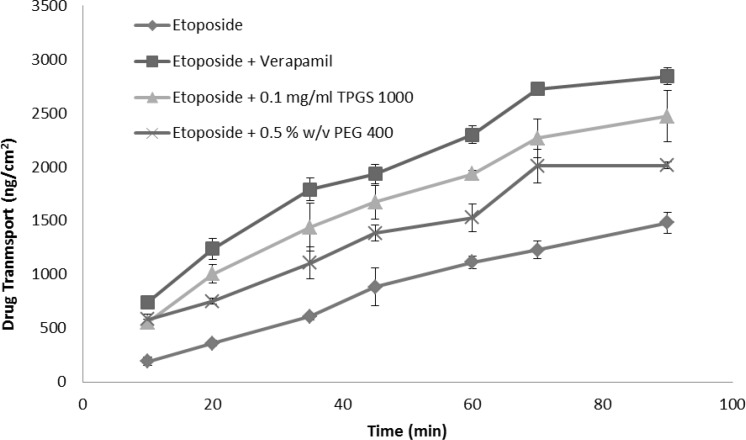
Intestinal absorption of etoposide (control) and etoposide in presence of verapamil, TPGS or PEG in everted gut sac model. Data are shown as mean ± SE (n = 3-5).

Verapamil, the most extensively characterized P-gp inhibitor, was used as a positive control and reference standard to compare the P-gp inhibitory potential of excipients (30). The time course of absorptive transport of etoposide across small intestinal segments in presence and absence of verapamil is illustrated in Figure 4. 

Our results showed that when the P-gp inhibitor, verapamil, was administered, etoposide absorption into the sac contents was markedly elevated, *i.e.*, the addition of verapamil at 100 μg/mL concentration led to significant increase of etoposide absorption in the sac content by 90% compared to control (Figure 4). Additionally the calculated permeability of etoposide with verapamil was higher than control by two folds (Table 1, p < 0.001). This result indicated that inhibition of P-gp induced by verapamil can markedly enhance the intestinal transportation and permeability of etoposide, and subsequently increase its bioavailability. Etoposide has a low bioavailability *in-vivo *not only due to its poor permeability and first pass metabolism; but also owing to the contribution of efflux transporters such as P-gp in the intestine (31). Accordingly the inhibition of P-gp transporters could be a strategy to increase permeation in the absorptive direction.

**Table 1:** Etoposide permeability in presence of verapamil, TPGS and PEG 400.

	**Permeability (s/cm)×10** ^-5^ **(mean ± SD)**	**p-value** **(compared to the control)**
Control (no excipients)	1.01 ± 0.09	
Verapamil (100 μg/mL)	2.03 ± 0.06	< 0.001
TPGS (0.1 mg/mL)	1.67 ± 0.20	< 0.05
TPGS (0.02 mg/mL)	1.96 ± 0.66	< 0.05
TPGS (0.002 mg/mL)	1.64 ± 0.25	< 0.05
PEG 400 (0.5% w/v)	1.33 ± 0.16	> 0.05
PEG 400 (0.1% w/v)	1.45 ± 0.33	> 0.05
PEG 400 (0.05% w/v)	1.42 ± 0.24	> 0.05

On the next step, effect of PEG 400 and vitamin E-TPGS 1000 on etoposide transport was evaluated and the calculated permeabilities are shown in Table 1. Etoposide absorption was not affected by all tested concentration levels of PEG 400 (p > 0.05), while TPGS in all tested concentrations had significant effects on etoposide permeability compared to the control. The highest drug permeability was obtained by TPGS 0.02 mg/mL (1.96 ± 0.66 ×10^-5^ s/cm) although it was not statistically different from the other TPGS concentrations (0.02 and 0.1 mg/mL).

There is contradicting reports about the inhibitory effect of PEG 400 on P-gp. Rege *et al. *observed no effect of PEG 400 on the transport of cimetidine and furosemide, drugs subject to the action of efflux transporters, across Caco-2 cells (32). On the other hand using rat intestine mounted in Ussing chambers, and digoxin as the P-gp substrate, Johnson *et al. *observed significant inhibition of efflux with PEG 400 (17). In another study Li and coworkers reported that PEG-400 could increase the transport amount of ganciclovir in the everted gut sac model (18). In our experiments no significant effect of PEG-400 on etoposide permeability was observed. With regard to TPGS, Johnson *et al. *failed to observe any P-gp inhibition effect (17), nevertheless the inhibitory effect of this compound on P-gp has been reported in several studies by using different methods (19, 23, 33, 34). Here we found TPGS very effective in enhancement of etoposide transport, more likely due to P-gp inhibition, even in very low concentration (0.002 mg/mL). This effect of TPGS was concentration independent and there was no considerable difference in etoposide permeability between the various concentrations of TPGS that were studied (*i.e. *0.002 to 0.1 mg/mL). Based on our results TPGS could improve the etoposide intestinal absorption and permeability and it would be a good choice as a non-toxic emulsifier for further *in-vivo *bioavailability studies (19).

*Effect of TPGS on paracelluar and transcellular transports*

To investigate whether TPGS can alter drug transport via the paracelluar route, the tissue was co-incubated with TPGS and lucifer yellow. Lucifer yellow is the marker used to study the paracelluar absorption along the small intestine (25).

No significant effect on the permeability of lucifer yellow was observed in presence and absence of TPGS 0.1 mg/mL. (9.65 ± 0.6 × 10^-6^ cm/s and 9.3 ± 0.03 × 10^-6^ cm/s, respectively). 

Afterward imipramine as a transcellular marker, was used to check the integrity of biomembrane (23). The permeability of imipramine was 1.14 ± 0.32 × 10^-5^ cm/s across the intestine membrane. By addition of TPGS (0.1 mg/mL) this result was not changed significantly (1.01 ± 0.27×10^-5^ cm/s) indicating that enhancement of etoposide permeability in the presence of various concentrations of TPGS were not due to injure of membrane integrity. 

In general several mechanisms could be suggested for enhancement of etoposide absorption by excipients including (a) increasing the solubility of hydrophobic drugs (35), (b) interaction with metabolizing enzymes such as CYP3A (36), (c) disruption of tight junctions (37, 38), (d) local damage of the intestinal epithelium (39). In addition, it is quite acceptable that inhibition of P-gp mediated drug efflux will enhance absorption of P-gp substrates. To investigate this mechanism, the other possibilities need to be excluded. Considering the applied concentration of TPGS in our studies which was below CMC (0.2 mg/mL) (23), the micelle formation and its effect on solubility of etoposide did not take place. The obtained results for the transport of lucifer yellow suggest that the paracelluar route, which is a passive diffusion mechanism for small hydrophilic molecules, was intact throughout the small intestine when TPGS was applied. Moreover, the results from the unchanged permeability of lucifer yellow and imipramine with TPGS indicates that the tight junctions and integrity of intestinal epithelium remained intact. Also the cytotoxic effect of the excipients on intestinal epithelium was disregarded by statistically equal data from obtained LDH activity with and without TPGS.

The metabolism of etoposide is mediated principally by CYP3A4 which is located in the intestine (40). The potential effect of TPGS on this metabolic rout of etoposide remains to be more evaluated. However, our results demonstrate that p-g-p inhibition by TPGS could be considered as the most likely mechanism for enhancement of etoposide absorption. This finding could be essential to be considered in drug formulation strategies using TPGS as a safe excipient.
